# The chemical composition of free-range and conventionally-farmed eggs available to Canadians in rural Nova Scotia

**DOI:** 10.7717/peerj.11357

**Published:** 2021-05-04

**Authors:** Marcia M. English

**Affiliations:** Human Nutrition, Saint Francis Xavier University, Antigonish, Nova Scotia, Canada

**Keywords:** Free-range eggs, Conventionally-farmed eggs, Gas chromatography, Cholesterol, Amino acid composition, Small-scale egg producers, Specialty eggs, Fatty acid composition, Protein

## Abstract

In rural Nova Scotia (NS), many small family farms raise free-range hens that consume a varied diet that is different from that of conventionally-farmed hens in caged housing systems. However, it is not known how this varied diet impacts the quality of these eggs. The objective of the present study was to compare the chemical composition of free-range eggs obtained from a small family farm in rural NS to that of conventionally-farmed eggs purchased from a local grocery store. The values obtained from the present study were also compared to published values in the Canadian Nutrient File (CNF) and the U.S. Department of Agriculture FoodData Central database. The egg components and the amino acid compositions were evaluated, and protein concentrations were determined using the Kjeldahl method whereas the fatty acid profiles of the egg yolks were determined using gas chromatography. No difference (*P* = 0.3) in protein content was observed in free-range eggs (10.6 ± 1.1%) compared to conventionally-farmed eggs (9.7 ± 0.6%). Similar values were also observed for the physical properties of the two types of eggs measured except for the weights of the egg shells. Conversely, the amino acid cysteine, was in higher amounts (*P* = 0.05) 0.26 g/100 g in the CNF compared to the measured values of ~0.16 g/100 g. Notably, the polyunsaturated linoleic acid (C18:2n-6) was higher (*P* = 0.001) in the free-range eggs (45.6%) compared to (40.8%) the conventionally-farmed eggs. The cholesterol content of egg yolks was lower in free-range eggs (253.4 ± 0.01 mg/extra-large yolk or 14 mg cholesterol/g of yolk) vs. for conventionally-farmed eggs (263 ± 0.7 mg/extra-large yolk or 15.4 mg cholesterol/g of yolk), respectively. In terms of protein nutrition, free-range eggs may be a suitable alternative to conventionally-farmed eggs, moreover, the lower cholesterol content may be a favourable attribute for Canadian consumers who wish to purchase local free-range eggs.

## Introduction

In Canada, eggs are an everyday food item found in most households, and they provide a convenient meal filled with protein, fat, vitamins, and minerals all contained in a perfectly formed natural container: their shell. The widespread use of eggs is as a result of a well-established egg industry; valued at about $1.4 billion dollars (Statistics Canada, 2019). In addition, eggs have many useful functional properties that make them extremely important in food production such as their ability to coagulate, aerate when whipped, and emulsify (Chang et al., 2018).

Currently, laying hens are housed in a variety of ways in Canada and include: commercial poultry farms, where birds live in cages in an environmentally controlled barn; furnished or enriched housing, where birds have more space (floor and height) and are able to roam around the floor within the barn, enrichments may include nesting boxes and scratching pads; and free-range, where birds have access to roam both indoors and out, provided the weather permits (Egg Farmers of Alberta, 2020). Among these methods, conventional housing constitutes about 90% of egg production, whereas free-run, organic, and free-range systems account for the other 10% (Egg Farmers of Canada, 2016a). However, recent consumer purchasing trends in Canada indicate an increased demand for specialty eggs such as organic and free-range products (Bejaei, Wiseman & Cheng, 2011; Statistics Canada, 2019). In addition, increased public demand for greater transparency with regard to production practices, and hen welfare has resulted in the initiation of a coordinated, systematic transition from conventional cage-based egg production towards alternate non-caged methods of egg production in Canada (Egg Farmers of Canada, 2016a).

On some family farms in rural Nova Scotia, Canada, small-scale egg production is based on systems in which free-range hens feed on materials from the surrounding environment including cultivated and wild vegetation, insects, and supplemented grains (Egg Farmers of Canada, 2016b; Krawczyk et al., 2013). This means free-range hens have much greater variation in their diet than conventionally-farmed hens that only consume standard chicken feed, which are usually wheat or corn-based (Egg Farmers of Canada, 2016b). Although the egg industry in Canada is slowly transitioning away from cage-based production (Pelletier et al., 2018), the current egg quality profile largely reflects the production of eggs under the conventional caged system (Canadian Nutrient File, 2015). This is an interesting observation since, since egg production under free-range systems depend to a large degree on the quality of the feed available for scavenging, which in turn impacts the quality of the egg (Krawczyk et al., 2013; Rizzi & Marangon, 2012). It is also well known that small, family egg farms not only supply their own needs but have also provided opportunities for income generation in their local communities (Zaheer, 2015). However, in order to grow their businesses, these small-scale egg producers need to differentiate their products in the marketplace. This clarity is also needed for consumers who purchase these commodities (Bejaei, Wiseman & Cheng, 2015).

Several studies have emphasized the ability of hens to transfer nutrients that they consume through their food into their eggs. For example, Hansen et al. (2015) explored the transferability of several forms of vitamin-E into the eggs of Hy-Line W-36 laying hens (30 weeks old), and showed that α-tocopherol was transferred efficiently from hen feed to their eggs. In a similar study, Yao et al. (2013), investigated the impact of feeding (vitamin-D3) cholecalciferol-enriched diets on the egg quality of Hy-Line W-36 hens (19 weeks old), and found that even the lowest feed concentration (2,200 IU/kg feed) contributed to an increase in the amount of vitamin-D. Notably, the majority of studies examining nutrient transfer in free-range hen’s eggs have focused on the impact on lipid composition or lipid-containing compounds. This trend was prompted in part by earlier perceptions that eggs were implicated in the development of chronic heart disease because they contain a significant amount of cholesterol (Djousse & Graziano, 2008; Li et al., 2013).

Conversely, eggs are considered to be of high biological value because of their high protein content (Chang et al., 2018). Moreover, in their 1973 study, Lunven et al. used the amino acid composition of eggs from White Longhorn and New Hampshire hens as a measure of their protein quality. Later, Lešić et al. (2017) also reported that eggs are a valuable source of protein because they contain essential amino acids. Yet, few studies have examined how egg production under a free-range system impacts protein content and amino acid composition. The aim of the present study was to provide a comparative baseline analysis between the physical and chemical properties of free-range and conventionally-farmed eggs that may be of interest to consumers and egg producers. Furthermore, this data was not previously available for egg products in rural Nova Scotia. Based on the current literature that emphasized the ability of hens to transfer nutrients from their diet to their eggs it was hypothesized that the chemical composition of the two types of eggs examined would be different. The chemical data obtained in the present study were also compared to the current data in the Canadian Nutrient File (2015) and the U.S. Department of Agriculture FoodData Central database. Indeed, this type of nutritional characterization may help small-scale egg farmers with the marketing of their products and will also help consumers make more informed purchasing decisions based on the nutritional quality of eggs.

## Materials & methods

### Egg collection and preparation

Two dozen free-range eggs were collected (September, 2015) from a small family farm in Antigonish, Nova Scotia (NS) and prepared as described below. For these experiments free-range hens were allowed to be in their natural environment, and their surroundings and diets were not controlled. Laying hens at the local family farm (19 weeks) were from the following varieties Black and Grey Australorp, and Rhode Island Red. The hens spent most of their time wandering around outside, laid their eggs in a chicken coop, and went inside a large ~9.1 × 15.2 m (30 × 50 ft) barn at night. The diet fed to hens included vegetable peels, scraps, lay mash, leftover bread, raw goat’s and cow’s milk, grass, insects, and worms.

On the other hand, two dozen fresh, conventionally-farmed white eggs were purchased from a local grocery store in Antigonish, NS. The diet of the conventionally-farmed eggs included a commercial feed composed of a blend of grains with ~15% protein, 1.5% fat and 4.5% fibre (multipurina.ca). The eggs were selected from the back of the grocery store fridge where the freshest eggs are kept, and also where the most consistent temperature is maintained. To access the physical properties of the eggs, whole eggs were weighed and then cracked open, following which intact yolks were separated using an egg separator. The total weights of egg yolks (grams) and eggshells were also recorded, and their albumen weights were calculated by difference (Samman et al., 2009; Lordelo et al., 2017).

### Extraction of protein

Proteins were extracted from the white component of both types of eggs as descried by Duan et al. (2013) with slight modifications. Twelve free-range eggs, and twelve conventionally-farmed eggs were used for the analysis. The eggs were weighed, cracked, and the whites were separated out from the yolks using an egg separator. The egg white samples were then defatted by washing them with 10 volumes (w/v) of cold acetone, which was approximately 500 mL of acetone for 50 mL of albumin. The solution was thoroughly mixed with a stir bar then left to dry overnight at 4 °C. The resulting acetone powder was agitated with phosphate buffered saline (PBS, pH 7.4) overnight at 4 °C. The extracted proteins were subsequently centrifuged at 2,500*g* for 15 min at 4 °C and then the supernatants were collected and centrifuged at 12,000*g* for 3 min at 4 °C. The protein samples were then lyophilized, and stored at −20 °C for further testing.

### Amino acid analysis

Amino analysis was performed at the Hospital for Sick Children, Peter Gilgan Centre for Research & Learning (SPARC BioCentre, Toronto, Canada). The analysis was performed using a Waters Acquity UPLC System (Milford, MA, USA). Egg white protein samples (0.01 g) were hydrolyzed with 6 N hydrochloric acid with 1% phenol and using the Waters Pico-Tag Workstation, air was removed from the reaction vial, replaced with pre-purified nitrogen and sealed under nitrogen. The reaction vials were placed in the block heater to hydrolyze for 24 h at 110 °C. After hydrolysis, the samples were dried by a Tomy CC-181 Centrifugal Concentrator with a Sargent-Welch Model 8821 vacuum pump and the excess HCl was also removed during this process. After drying, the samples were treated with a redrying solution consisting of methanol: water: triethylamine (2:2:1), then mixed and dried under vacuum for 15 min.

The samples were then derivatized for 20 min at room temperature with a derivatizing solution made up of methanol: water: triethylamine: phenylisothiocyanate (PITC) (7:1:1:1). After 20 min, the derivatizing solution was removed under vacuum and the samples were dissolved in a sample phosphate buffer (pH 7.4) and an aliquot (4 μL) injected into the column (UPLC BEH C18, 2.1 × 100 mm), running on a modified PICO- TAG gradient. The column temperature was 480 °C and the derivatized amino acids were detected at 254 nm using a TUV Detector Module. Data were collected and processed using Waters Empower 3 Chromatography software.

### Protein determination

The crude protein contents of the egg white samples were determined using the American Association of Agricultural Chemists (AOAC) International Kjeldahl method as described by Duan et al. (2013). To digest the protein samples, the following components were added to labelled digestion tubes, 0.5 g of dried protein, two catalyst tablets (MT-37 Kjeldahl tablets; Fisher Scientific, Waltham, MA, USA), 10 mL of Kjeldahl acid, and 5 mL of 30% H_2_O_2_. The tubes were then placed in a preheated digestion block (DK20; VELP Scientifica, Usmate Velate MB, Italy) set at 420 °C, and the samples were digested for 20 min. The samples were cooled for 5 min, and subsequently diluted with 70 mL of water.

To distill the samples, 10 mL of sodium thiosulfate was added to the diluted samples immediately following the digestion. A 250 mL Erlenmeyer flask containing 25 mL of 2% boric acid solution (w/v) and a couple drops of indicator was placed under the condenser outlet, and 50 mL of 40% NaOH was slowly dispensed. The steam distillation was then conducted. The distillate was collected then titrated with 0.199 M HCl. The entire process was repeated three times for both types of eggs. The following equation was used to calculate the percent protein in the egg samples, where a nitrogen conversion factor (*f*) of 6.25 was used to quantify the protein content.

$$\%\;Protein=\frac{\left(mL\;HCl\;for\;sample-mL\;HCl\;for\;blank\right)\;\times \;Molarity\;\times \;f\;\times \;14\;\times \;100}{mg\;of\;sample}$$

### Fatty acid determination

Fatty acid determination was adapted from the method by Bannon et al. (1982). Twelve fresh free-range and conventionally-farmed eggs were weighed and the yolks were separated manually using a kitchen egg separator. The yolks were pooled and homogenized (VWR Scientific Vortex Genie; Marshall Scientific, Hampton, NH, USA) for one min, after which 15 mL of yolk was mixed with 25 mL of a chloroform: methanol (2:1, v/v) blend. Each sample was then prepared by adding to labelled 20 mL vials, 1 mL of hexane and 3 mL of the egg yolk, chloroform: methanol blend. The vials were then shaken, and allowed to sit for 10 min before centrifuging at 3000×*g* for 15 min. Two grams of anhydrous sodium sulphate (Fisher Scientific, Waltham, MA, USA) were added to the collected supernatants, and after mixing, the tubes were left to stand for 10 min. Base-catalyzed transesterification was carried out by transferring 30 μL of the supernatant to a 2 mL vial and adding 50 μL hexane and 100 μL of a methylation solution (sodium methoxide in methanol, 25% w/v). Transesterification was carried out at room temperature for 20 min. All procedures were performed in triplicate for the two types of egg samples.

For gas chromatography analysis, 1 mL of each transesterified sample was diluted to 10 mL with pure hexane, and subsequently filtered (0.22 μm) before being placed in 2 mL vials. Hexane (0.5 mL) was added to each vial before they were capped and loaded on the gas chromatograph (7890A; Agilent, Santa Clara, CA, USA). A high polarity Agilent DB-23 (50%-Cyanopropyl-methylpolysiloxane) column; 30 m × 0.25 mm i.d., 0.25 µm was used. The Flame Ionization Detector (FID) was set at 260 °C, and the oven temperature was programed from 190 °C to 250 °C at 10 °C/min and held there for 10 min. Helium carrier gas was used and the flow rate was set at 1.5 mL/min. The values obtained in the present study for the protein and lipid profiles were also compared with the current values in the Canadian Nutrient File (CNF), https://food-nutrition.canada.ca and the USDA FoodData Central database (https://fdc.nal.usda.gov/).

### Cholesterol extraction

Cholesterol extraction was adapted from the method outlined by Botsoglou et al. (1998). Egg yolks were weighed and separated as described for the lipid extraction. Then 0.2 g of the homogenized samples were added to 5 mL of methanolic KOH (0.5 M) and after mixing the samples were placed in a water bath (Isotemp; Fisher Scientific, Waltham, MA, USA) set to 37 °C for 15 min. The samples were vortexed every 5 min following which they were cooled under running tap water. One milliliter of distilled water and 5 mL of hexane (Sigma Grade) were added to each tube, and the samples were centrifuged for 2 min at 2,000×*g*. The supernatants were removed, and 2 g of anhydrous sodium sulphate (Fisher Scientific, Waltham, MA, USA) were added to each tube, and the samples were left to stand for 10 min. The extraction was carried out in triplicate for the two types of egg samples.

For gas chromatography, 1 mL of each sample was diluted to 10 mL with pure hexane, and subsequently filtered (0.22 μm) before being placed in 2 mL vials. Each vial was capped and loaded on the gas chromatograph (7890A; Agilent, Santa Clara, CA, USA). A BR-1ms 30 cm × 0.25 mm 0.25 µm df (film thickness) column was used, and the injector and the detector temperatures were set at 300 °C. The initial oven temperature was set at 230 °C, and ramped up 1.5 °C/min to 270 °C and held there for 10 min. Helium carrier gas was used and the flow rate of 1.5 mL/min.

When reporting the amount of cholesterol in an egg, the weight size class of the egg should be specified Hansen et al. (2015). Based on the egg weight classification from the CNF (extra-large egg ~58.8 g) the two types of eggs were classified as extra-large. Then using the average weight of the egg yolks, and the amount of cholesterol (mg/00 g of yolk) measured, the cholesterol contents were reported as mg/g of extra-large yolk as described by Hansen et al. (2015).

### Statistical analysis

The results of the protein concentration are presented as mean and standard deviation of three independent experiments. Unpaired student’s *t*-tests were used to determine significant differences in the physical properties of the eggs, their amino acid compositions and protein concentrations as well as their fatty acid compositions and cholesterol contents. In all analyses, *P* values < 0.05 were considered significant. All data were analyzed using GraphPad Prism version 8.4.1 for MacOS (GraphPad Software, San Diego, CA, USA), www.graphpad.com.

## Results

### Physical properties

The physical properties of the two types of eggs are shown in Table 1. No significant difference in total egg weights were observed between free-range eggs (65.5 ± 9.0 g) compared to conventionally-farmed eggs (58.5 ± 1.5 g) (*P* = 0.11). Average albumen weights were higher in free-range and conventionally-farmed eggs (39.3 ± 7.4 and 33.6 ± 1.3) compared to the weights recorded for the yolks (17.4 ± 2.1 and 17.1 ± 1.2). However, no significant differences were observed for these components between the two types of eggs. Similar trends were also observed for albumen pH. Conversely, there was a significant difference in the weight of the shells measured for free-range eggs (8.8 ± 0.3 g) vs. conventional eggs (7.8 ± 0.2) (*P* = 0.002). However, when the shell weights were expressed as a percentage of total egg weights these differences were no longer significant between the two types of eggs.

**Table 1:** Mean physical egg properties of free-range and conventionally-farmed eggs.

Egg components	Free-range eggs	Conventional eggs
Egg wet weight (g)	64.6 ± 9.0	58.5 ± 1.5
Yolk (g)	17.4 ± 2.1	17.1 ± 1.2
Yolk% (percentage of egg weight)	26.7 ± 2.2	29.3 ± 1.7
Albumen (g)	39.3 ± 7.4	33.6 ± 1.3
Albumen% (percentage of egg weight)	57.9 ± 3.3	57.4 ± 1.7
Albumen pH	8.8 ± 0.4	9.1 ± 0.04
Shell (g)	8.8 ± 0.3^a^	7.8 ± 0.2^b^
Shell% (percentage of egg weight)	13.6 ± 1.6	13. 4 ± 0.2

**Note:**

The values are represented as means ± SD. Values with different letters in a row are significantly different (*p* < 0.05). The data represent average values from *n* = 24, free-range and conventionally-farmed samples.

### Chemical components (protein and lipid profiles)

#### Protein profile

To access the protein quality of free-range eggs and conventionally-farmed eggs, their amino acid compositions were measured. These values, and how they compared to the data in the Canadian Nutrient File (CNF) are shown in Table 2. Overall, the amino acid contents from the two types of eggs were comparable, and aspartic acid was the most abundant amino acid in both types of eggs, with registered values of 1.61 g/100 g for free-range eggs vs. 1.5 g/100 g for conventionally-farmed eggs. Conversely, minimal differences were observed between the measured values in the present study compared to the amino acid data in the CNF. For example, the measured values for leucine were similar for free-range eggs (0.72 g/100 g) and conventionally-farmed eggs (0.70 g/100 g), whereas the value from the CNF was 0.92 g/100 g (*P* = 0.05). A similar trend was also observed for methionine, a higher value (*P* = 0.05) was observed in the CNF (0.39 g/100 g) compared to the registered values in both types of eggs, 0.2 g/100 g (Table 2). However, in the case of threonine, the data obtained in the CNF (0.47 g/100 g) was lower than the values measured for free-range eggs (0.65 g/100 g) and conventionally-farmed eggs (0.64 g/100 g) in the present study.

**Table 2:** Chemical composition (protein concentration and amino acid composition) of egg white and the cholesterol contents of yolks obtained from free-range and conventionally-farmed eggs. The average weight of the egg whites and the egg yolks are also shown.

Names of amino acids	Free-range eggs (g/100 g)	Eggs from conventionally-farmed hens eggs (g/100 g)	Data from Canadian nutrient file (g/100 g)
***Non-essential***			
Alanine	0.45	0.44	0.66
Aspartic acid	1.61	1.54	1.14
Cysteine	0.16	0.15	0.26
Glutamine	1.13	1.10	1.47
Glycine	0.43	0.42	0.38
Proline	0.38	0.37	0.40
Serine	0.56	0.55	0.75
***Essential***			
Arginine	0.54	0.53	0.61
Histidine	0.36	0.35	0.26
Isoleucine	0.26	0.25	0.57
Leucine	0.72	0.70	0.92
Lysine	0.83	0.78	0.75
Methionine	0.20	0.18	0.39
Phenylalanine	0.44	0.42	0.66
Threonine	0.65	0.64	0.47
Tyrosine	0.51	0.49	0.43
Valine	0.72	0.69	0.75
**Chemical Composition**	**Free-range Eggs**	**Conventional Eggs**	**CNF Data**
**Egg white: Total protein**^c^	10.6 ± 1.1%	9.7± 0.6%	11.8%
**Average total weight**	65.5 ± 9.0 g (extra-large)	58.5 ± 1.5 g (extra-large)	56 g (extra-large)
			**USDA Food Data Central**
**Egg Yolk:**^c^ Cholesterol content	253.4 ± 0.01^a^ (mg/extra-large yolk)	263 ± 0.7^b^ (mg/extra-large yolk)	210 (mg/extra-large yolk)
Average weight of yolk^c^	17.4 ± 2.1 g	17.1 ± 1.2 g	17 g

**Note:**

a Food codes from the CNF: whole, fresh, raw egg (code 125); and egg white (code 126).

b Food codes from the USDA FoodData Central: Grade A extra-large eggs, 56 g (cholesterol value).

c Values for average total weights, egg yolks and cholesterol values are for the means and standard deviations for (*n* = 12).

The egg white samples were also characterized by measuring their total protein concentrations, and no statistically significant difference (*P* = 0.3) was noted between the measured values for the two types of eggs (Table 2). Free-range eggs registered values of 10.6 ± 1.1% compared to 9.67 ± 0.6% in conventionally-farmed eggs. The measured values in the present study were also similar to the protein concentration (11.8%) reported in the CNF.

### Lipid profile

**Figure 1:**
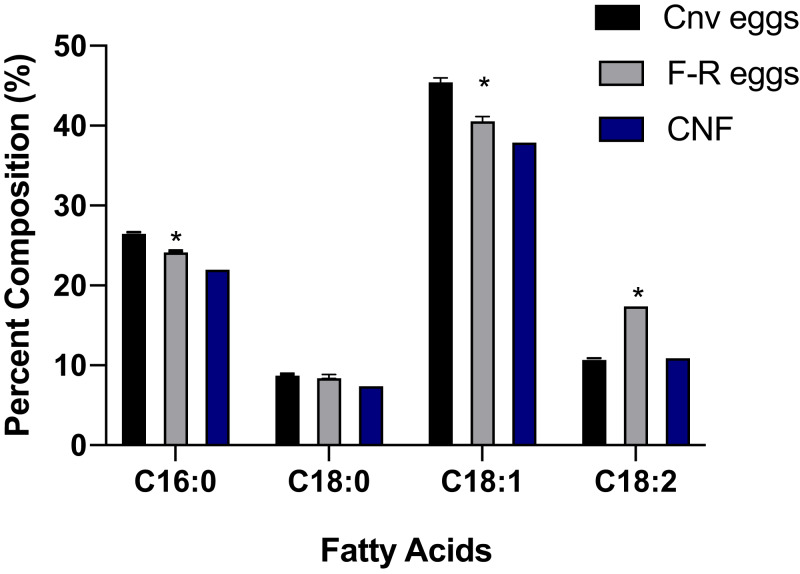
The percent fatty acid (FA) composition of free-range eggs from a local family farm vs. conventionally-farmed eggs. The measured values in the present study were compared to the published values in the Canadian Nutrient File (CNF). The main FAs represented are palmitic acid (C16:0), stearic acid (C18:0) oleic acid (C18:1n-9), and linoleic acid (C18:2n-6). The FA compositions were determined using gas chromatography. Unpaired student’s t-tests were used to determine significant differences in the fatty acid compositions of the different types of egg samples. In all analyses, *P* values < 0.05 were considered significant which are indicated by an asterisk (*).

To understand the relationship between the varied diet in free-range hens and their lipid composition, the relative abundance of the main fatty acids (FAs) present in their egg yolks was measured. These values and how they compared to the profile in conventionally-farmed eggs, and data in the CNF are shown in Fig. 1. In the present study, the measured values for the most abundant FAs ranged from 8.6% to 45.6%. In both types of egg yolks, the monounsaturated FA, oleic acid (18:1n-9) was the most abundant in free-range eggs, 45.6% vs. 40.8% in conventionally-farmed eggs (*P* = 0.01). The saturated FA, palmitic acid (16:0) was second in relative abundance, with 24.3% registered for free-range eggs whereas the concentration was significantly higher (*P* = 0.04) in conventionally-farmed eggs (26.7%). Significant differences (*P* = 0.005) were also observed in the amounts of the polyunsaturated FA, linoleic acid (18:2n-6) in the free-range eggs, 17.6% vs. the 10.9% registered in conventionally-farmed eggs. The latter (10.9%) was also more similar to the value reported (11.1%) in the CNF.

Similar to the FA profile, significant differences (*P* = 0.05) were also observed in the cholesterol content of both types of eggs (Table 2). Although the average weight of the yolks was similar for both types of eggs (Table 2), the cholesterol content was greater (*P* < 0.05) in conventionally-farmed eggs (263.0 ± 0.7 mg/extra-large yolk or 15.4 mg cholesterol/g of yolk) compared to the free-range eggs (253.4 ± 0.01 mg/extra-large yolk or 14 mg cholesterol/g of yolk). The calculated cholesterol values were both higher than the value reported in the USDA FoodData Central database (210 mg/extra-large yolk or 10.8 mg cholesterol/g of yolk).

## Discussion

This work was focused on providing a baseline comparison of the physical and chemical properties of free-range eggs obtained from a small family farm vs. conventionally-farmed eggs in Nova Scotia, Canada. The measured values were also compared to the current data in the Canadian Nutrient File (CNF) and the USDA FoodData Central database.

Except for the significant differences observed in the weights of the egg shells, similar values were obtained for the other physical properties measured in the present study (Table 1). In the egg industry, albumen pH is used as an indicator of egg quality and freshness (Silversides & Scott, 2001). It is believed that as eggs age, carbon dioxide is lost through the shell and the contents of the shell become more alkaline (Lordelo et al., 2017). Although no difference in albumen pH was observed in the egg samples in the present study, the eggs evaluated showed a higher albumen percent compared to the yolk. In addition, the weights of the shells from the free-range eggs were heavier (*P* = 0.002) than those from the conventionally-farmed eggs. However, these differences were no longer significant when expressed as a percentage of total egg weight. This finding agrees with the study by Lordelo et al. (2017) who also observed no difference in the percentage of shells among free-range eggs when compared to other labelled eggs. These data were not available for comparison with the information in the CNF. Changes in the proportion of egg components have been attributed to the age and the strain of the hen (Akbar et al., 1983). Although these physical properties may not be as important to consumers, partly because eggs are graded and defects are removed, the former are important attributes that are used to evaluate the quality of eggs in the food industry (Fletcher et al., 1981; Molnár et al., 2016).

On the other hand, knowledge of the chemical composition of eggs has not only been of interest to consumers and egg producers but has also been the focus of recent studies (Bejae et al., 2011; Abdou, Kim & Sato, 2013; Lordelo et al., 2017). In the present study, similar protein concentrations were observed in the free-range and conventionally-farmed eggs. This observation is consistent with the majority of the limited literature that refer to the protein quality of hen’s eggs (Duan et al., 2013). However, contrary to this, Hidalgo et al. (2008) did find a small but statistically significant difference (*P* < 0.05) in the protein content of eggs that came from different housing systems in Italy. Using the Kjeldahl method it was determined that caged hens produced eggs with 12.1 ± 0.09 g/100 g of protein whereas free-range hens produced eggs with 12.5 ± 0.20 g/100 g of protein. It is well known that conventionally-farmed hens eat a diet of mostly grains, protein, fats and minerals (Egg Farmers of Canada, 2016b). On the other hand, hens in the present study had a varied diet which included milk (cows’ milk, 3.28 g/100 g protein *and* goats’ milk, 3.56 g/100 g of protein), insects 40 to 75 g/100 g of dry weight (Verkerk et al., 2007), and a commercial feed. The latter, Purina Purinature Layena Checkers Laying Complete, contained 17% protein for every 100 g of feed. However, this rich protein diet in free-range hens did not improve their protein concentration (Table 2) and the only changed observed in their physical components was the weights of their egg shells (Table 1).

In an earlier study, Lunven et al. (1973) demonstrated that different breeds of hens (White Leghorn vs. New Hampshire) receiving varied quantities of dietary proteins (110 vs. 200 g protein/kg for 12 months) produced eggs with no significant changes in their amino acid compositions. The measured data in the present study is consistent with this observation but does not support the original hypothesis that the varied diet of free-range hens would impact the amino acid compositions of their egg white proteins. Thus, at least where protein nutrition is concerned, free-range eggs may be a suitable alternative to conventionally-farmed eggs. Conversely, when the measured values in the present study were compared to the data in the CNF, some variations in the amino acid compositions were observed (Table 2). For example, the sulfur-containing amino acid cysteine, was in higher amounts (*P* = 0.05) 0.26 g/100 g compared to the measured values of ~0.16 g/100 g (Table 2). The smell of a cooked egg comes from a reaction between iron in the egg yolk and sulfur in the egg white, forming ferrous sulfide (Vaclavik & Christian, 2014). Thus, when cysteine is present in higher quantities there may be an increase in the strength of the aroma, however, specific sensory analyses would be required to investigate this hypothesis.

Although a firm conclusion cannot be made about what might have caused the differences observed in the amino acid compositions in the measured data in the present study and the data in the CNF, it is reasonable to assume that differences in the analytical methods used could be a driving factor. The amino acid compositions in the present study were determined using the chromatography approach outlined previously in the methods. Although a similar approach may have been used to determine the amino acid composition in the CNF database, the specific amounts of egg-white proteins used, and the hydrolysis procedures are unknown. Indeed, some authors believe that errors in the hydrolysis step is primarily responsible for variations in the determined compositions (Fountoulakis & Lahm, 1998). Moreover, no single hydrolysis method exists for all residues, thus, hydrolysis may represent a limiting step in amino acid composition determination (Fountoulakis & Lahm, 1998).

To further characterize the chemical composition of the eggs in the present study, their fatty acid profiles were evaluated (Fig. 1). In both types of eggs examined, oleic acid (C18:1n-9) was the major FA registered confirming the observations of Cherian, Holsonbake & Goeger (2002). However, the relative abundance of linoleic content reported in the present study was higher in free-range eggs (17.6%) compared to the registered value for conventionally-farmed eggs in the present study (10.6%) and the value (16.2%) reported by Cherian, Holsonbake & Goeger (2002). However, in this instance, the data from the CNF (10.9%) was more comparable to the measured data from conventionally-farmed eggs (Fig. 1). The higher content of the polyunsaturated linoleic acid (18:2n-6) in free-range eggs from the local farm may be beneficial for their consumers since linoleic acid is an essential fatty acid, meaning it must be supplied from the diet (Whelan & Fritsche, 2013). Linoleic acid is also an important structural component of the membrane phospholipids. This FA also acts as the parent compound for the omega-6 polyunsaturated FA, including arachidonic acid (20:4n-6) which can be converted to other cellular mediators with important normal metabolic functions (Whelan & Fritsche, 2013).

Although the average weight of the yolks was similar for both types of eggs (17.3 ± 0.9 g), the cholesterol content was greater in conventionally-farmed eggs (263.3 ± 0.7 mg/extra-large yolk or 15.4 mg cholesterol/g of yolk) compared to the free-range eggs (243.6 ± 0.1 mg/extra-large yolk or 14 mg cholesterol/g of yolk). This suggests that the variable diets of the free-range eggs may not be the only reason for the differences in lipid composition observed in the present study. Indeed, Zita, Jeníková & Härtlová (2018) demonstrated that several factors including the hen breed and housing system can impact the cholesterol content of egg yolks. In addition, the measured cholesterol values in the present study were both higher than the value reported in the USDA FoodData Central database (210 mg/extra-large yolk or 10.8 mg cholesterol/g of yolk). The difference in cholesterol values may also be due to differences in the size of the egg yolks in the published USDA data *vs*. the measured data.

On the other hand, Anderson (2011) compared the nutrient content of free-range eggs vs. eggs produced by caged hens and found that there was no effect on cholesterol levels. Importantly, Hansen et al. (2015) emphasized the importance of specifying the weight size class (small, medium, large, extra-large) of the egg when reporting the cholesterol content. Using the CNF database, the weight of an extra-large egg is ~58.8 g whereas that of a large egg is ~52.8 g. In the present study, the average weights registered for the free-range eggs and conventionally-farmed eggs were 65.5 ± 9.0 g and 58.5 ± 1.5 g, respectively (Table 2). Accordingly, these eggs were regarded as extra-large. However, comparative cholesterol values for extra-large eggs were not available in the CNF, so the cholesterol values obtained in the present study were compared to the reported values in the U.S. Department of Agriculture FoodData Central database. For extra-large eggs (~56 g), the cholesterol content reported in the USDA FoodData Central database was 210 mg compared to 253.4 ± 0.01 mg and 263 ± 0.7 mg for free-range and conventionally-farmed eggs, respectively. Given the variability observed in the weight of the eggs, it is not surprising that the cholesterol content reported in the USDA FoodData Central database, was less than the calculated values registered in the present study. Indeed, Fletcher et al. (1981) reported that increasing yolk yields were equally dependent on increasing egg weight. Since, cholesterol is present in the egg yolk, this finding suggests that larger eggs with larger egg yolks may contain higher cholesterol levels.

The results of the present study are limited by the inability to control several variables including the types of hens used in the study, and the type of commercial feed provided to the free-range hens. If these variables were controlled it would have altered the activities of the local farm and the method of raising their hens. Nevertheless, the study was meant to provide a comparative baseline analysis between the protein and lipid composition of free-range and conventionally-farmed eggs, since this data was not previously available for egg products available in rural Nova Scotia.

In addition, since other factors including their sensory preference drive the purchasing behaviour of consumers (Bejaei, Wiseman & Cheng, 2011), the current study highlights a potential focus to evaluate consumer preference of the two types of eggs. Specifically, it would have been interesting to conduct sensory evaluations to determine whether the differences in cysteine concentration would affect the aroma of the two types of cooked eggs. Furthermore, financial restrictions can also impact the decisions made in the grocery store. Specialty eggs are usually sold for a more expensive price ($4.94 Canadian dollars, CAN per dozen) than conventionally-farmed eggs ($2.74 CAD, per dozen) reportedly due to higher farm-level costs for non-cage egg production systems (Agriculture & Agri-Food Canada, 2020; Sumner et al., 2008). Although the increased price may not translate to additional protein benefits, the lower cholesterol content may be a favourable attribute for Canadian consumers who wish to purchase local free-range eggs.

## Conclusions

Overall, both types of eggs had similar physical properties, except for differences in the weight of the egg shells. For the chemical characterization, no difference in the protein quality of free-range eggs was observed when compared to conventionally-farmed eggs, confirming that the variable diet of free-range hens did not impact the protein quality of their eggs. The protein data in the CNF was also consistent with the measured values for protein concentration, however, there was some variability with the amino acid composition. Although the current study was limited by its inability to control the types of hens used and the type of commercial feed provided to the free-range hens, it highlighted differences between the fatty acid composition and cholesterol content of free-range and conventionally-farmed eggs available to Canadians in rural NS. Although no specific conclusions could be made about all the potential factors (types of hen and housing) that contributed to the differences observed in the lipid quality of the free-ranges eggs, it is reasonable to propose that the impact of the diet of the hens cannot be ruled out. The results of this study will allow small egg producers in rural NS to better understand their product. Moreover, this additional nutritional information may fuel new, accurate, product-specific marketing endeavors by small-scale egg producers which in turn may further increase consumer interests in these food commodities.

## Supplemental Information

10.7717/peerj.11357/supp-1 Supplemental Information 1 Supplemental data with amino acid profile of extra large eggs.

10.7717/peerj.11357/supp-2 Supplemental Information 2 Supplemental data for physical and chemical analyses.

## Additional Information and Declarations

### Data Availability

The following information was supplied regarding data availability: The raw lipid and protein data and the CNF with the amino acid composition are available in the Supplemental Files.
